# Dural Injury Treatment with a Full-Endoscopic Transforaminal Approach: A Case Report and Description of Surgical Technique

**DOI:** 10.1155/2022/6570589

**Published:** 2022-03-15

**Authors:** João Paulo Machado Bergamaschi, Fernando Flores de Araújo, Thiago Queiroz Soares, Kelsen de Oliveira Teixeira, Luiz Henrique Dias Sandon, Ricardo Graciano Squiapati, Gustavo Vitelli Depieri, Ariel Falbel Lugão, Rangel Roberto de Assis, Esthael Cristina Querido Avelar Bergamaschi

**Affiliations:** ^1^Clínica Atualli Spine Care-São Paulo, São Paulo, Brazil; ^2^Hospital Samaritano de São Paulo, United Health Group, São Paulo, Brazil

## Abstract

**Introduction:**

The objective of this study was to describe a surgical technique that uses transforaminal full-endoscopic access, which is different from the existing protocol, and to demonstrate another method of dural tear repair during endoscopic spine surgery.

**Background:**

Endoscopic spine surgery was initially described for lumbar disc pathologies. Technical advances and new materials have made it possible to treat cervical and thoracic spinal degenerative disorders. These advances have also made it possible to treat surgical complications, notably dural tears with CSF fistulas. The literature indicates that the incidence of these injuries ranges from 1% to 17%.

**Materials and Methods:**

Descriptive technical note of innovative and improved endoscopic surgical procedure exemplified with illustrative clinical case and comparative literature review.

**Results:**

There is only one report describing a full-endoscopic suture technique for dural sac repair. The gold standard for treatment of the most significant nonpunctate lesions continues to be a conversion to open surgery for lesion closure. Conversion can be problematic because most surgeries are performed under sedation and local anesthesia.

**Conclusions:**

In this case report and the new endoscopic suture technique described here, we show that primary correction of dural tears through endoscopy is possible. In addition to representing a paradigm break in solving one of the main complications of these procedures, it can expand the possibilities of spine endoscopy.

## 1. Introduction

Endoscopic spine surgery has gained increasing importance in the treatment of spinal pathologies. Initially described for lumbar disc pathologies, advances in materials and techniques have made it possible to broaden the scope of diseases that can be addressed [1, 2]. As such, it is possible to treat bone pathologies, such as foraminal and vertebral canal stenoses, along with pathologies of the cervical and thoracic spine [3–6].

This perspective of treating more complex diseases using an endoscopic route makes it possible to treat complications that may occur during the procedure, including dural tears with cerebrospinal fluid (CSF) fistulas [7, 8].

The incidence of dural sac injuries during lumbar decompression surgery is highly variable, ranging from 1% to 17% as reported in the literature [9–12]. When present, these lesions can be small and punctate. Repairs are unnecessary; however, more complex, larger lesions may occur, which require repair [13]. Until recently, the literature lacked descriptions of endoscopic techniques for repairing these lesions. Conversion to open surgery and repair in the traditional manner is usually recommended [14].

Shin et al. [15] described the first endoscopic lesion repair technique on the dural sac using the traditional uniportal microendoscopic technique. Park et al. [16] later described techniques for repairing lesions using biportal endoscopy.

The aim of this study was to report a case of dural tear sutured through transforaminal full-endoscopic access and to describe the surgical technique.

## 2. Case Report

A 56-year-old male patient presented with pain in the low back and right lower limb (L5 distribution), with hypoesthesia at L5 on the right side. The patient previously underwent endoscopic surgical treatment by transforaminal access at L4-L5 on the right for discectomy and decompression of the lateral recess, with recurrence of symptoms after eight months. Magnetic resonance imaging revealed new right centrolateral and foraminal disc herniation. Given the recurrence of symptoms, a new transforaminal endoscopic procedure was indicated for discectomy and decompression of the right lateral recess. The procedure was performed with the patient in a prone position with a 45° flexion of the hips and knees. Local anesthesia and sedation were administered. The same entry point as that in the previous surgery (approximately 11 cm from the midline) was used. An endoscope with a 4.2 mm working channel and 30° angulation was used. Intraoperatively, fibrosis was visualized in the foraminal and periradicular regions. During foraminoplasty and removal of part of the fibrosis from the right lateral recess, a longitudinal dural lesion measuring approximately 1.2 cm was observed.

## 3. Surgical Technique

The transforaminal endoscopic suture technique of the dural lesion was divided into three stages:

1. Preparation of the lesion region
2. Needle passage through the lesion
3. Suture and knot tightening

In the face of a dural tear, it is recommended that the pressure of the serum pump should not be increased, and additives (anesthetic, adrenaline, antibiotics, etc.) should be removed from the irrigation serum. We do not recommend the use of additives during any spinal endoscopic procedure. After the lesion is identified, it is essential to expand the surgical field to facilitate suturing. Through the transforaminal endoscopic access, an enlargement of the foraminoplasty was performed, including partial removal of the bone (superior articular process of L5 and cranial part of the L5 pedicle), the yellow ligament of the right lateral recess, the bulging disc of the foramen and right lateral recess, and part of the fibrosis around the lesion. This enlargement allowed for small movements of the working cannula, making it possible for the needle to pass through the lesion.

A 5–0 Prolene thread with two needles was used. One of the needles must be passed through the working channel of the endoscope to the injury site. Using a delicate, angled disc clamp, the needle was passed through one of the edges of the lesion and subsequently to the other edge. The method of picking up the needle with forceps may vary according to the location and type of injury.

**Figure 1:**
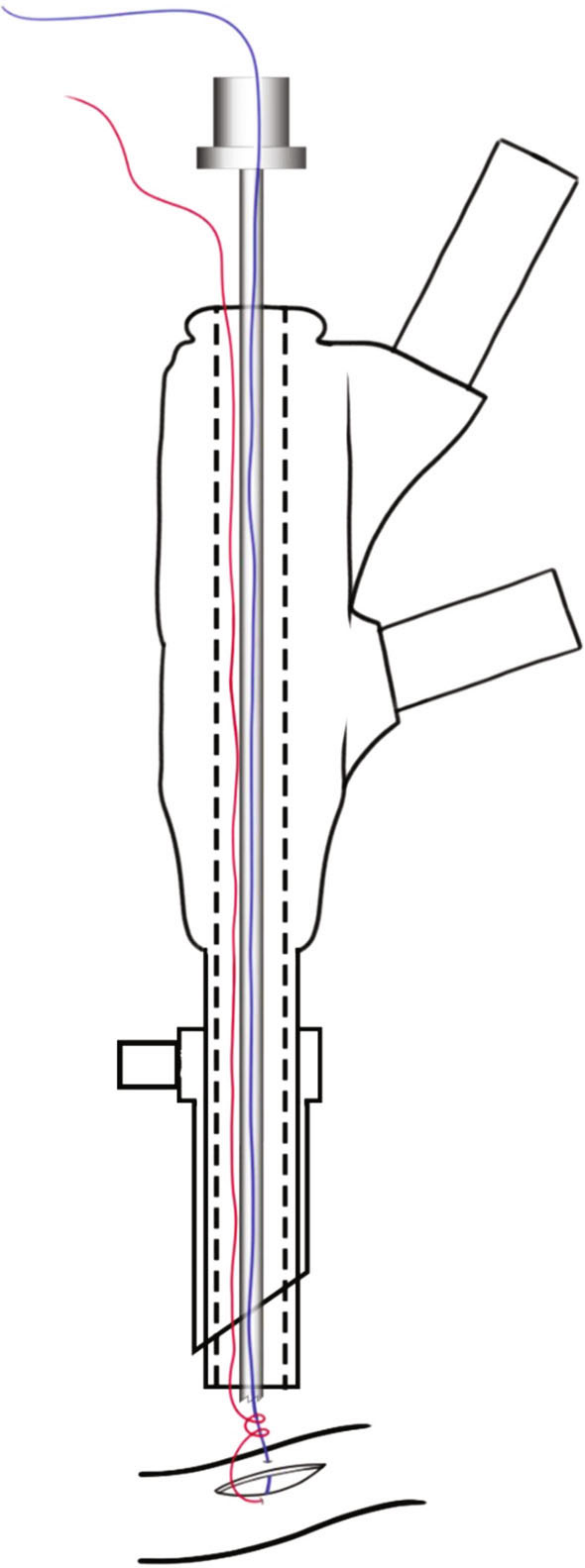
Schematic of the sliding knot using the endoscopic curette. Both threads pass through the working channel of the endoscope. After removing the needle, one of the threads (blue) passes through the endoscopic curette to move the sliding knot to the edge of the lesion.

**Figure 2:**
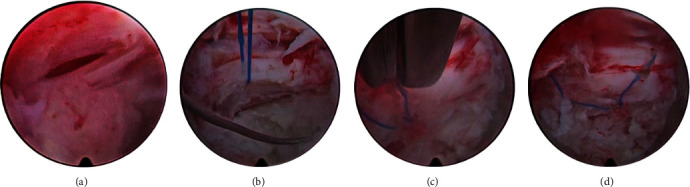
(a) Longitudinal dural tear of approximately 1 cm. (b) Thread passed through the two edges of the lesion. (c) Threads cut with endoscopic scissors after tightening the knot. (d) Final aspect of the suture.

After the needle was passed through the two edges of the lesion, the two ends of the thread were externalized to make the sliding knot. The execution of this knot allows it to slide to the lesion site without the need to pull the two ends of the wire. A curette was used to push the node to the lesion site under a direct view of the endoscope (Figure 1). Any other cannulated, noncutting material can be used for this purpose. It is important to note that at this stage, the wire must be pulled very carefully to avoid further damage to the dura. Finally, the ends of the wire were cut close to the knot using endoscopic scissors (Figure 2).

After suturing, discectomy and decompression procedures were completed without complications. The patient recovered without symptoms of CSF fistula and was discharged six hours after the procedure.

## 4. Discussion

With the rapid and constant evolution of surgical techniques and materials, endoscopic spine surgery is already moving toward becoming the gold standard in the treatment of disc pathologies. Muthu et al. assessed the superiority of the technique, establishing that endoscopic discectomy has superior outcome measures in factors such as the Oswestry Disability Index, surgery duration, complication rate, and length of hospital stay. It did not show inferiority in the other analyzed measures [17]. With the early adoption of the technique, it is natural that the complication rate increases because of its long learning curve [18].

With the popularization of this technique, there is also a need to develop techniques for the treatment of complications such as dural injuries. The incidence of dural tears varies between 1% and 17% in lumbar decompression surgeries [9–12]. In open surgeries, the indicated treatment is the primary repair of the lesion, considered by some authors to be essential for the prevention of CSF fistulas [11, 12]. Other techniques involve the use of fat, muscle, or artificial materials to promote occlusion of the dural lesion. They can also be associated with the primary suture of the lesion, mainly with fibrin glue [19–21]. Oertel et al. described the use of an autologous muscle graft interposition with the associated use of dural sealant as an effective way to repair dural sac injury, without performing a suture [22]. Conversely, Ahn et al. demonstrated that occlusion/interposition repairs without suturing through endoscopy are ineffective, recommending conversion to open surgery and direct injury repair [14].

In endoscopic spine surgery, dural lesions are most often characterized by small punctate lesions without large sequelae. Expectant treatments or occlusion with sealants, such as fibrin glue, may be recommended [13]. However, in the treatment of more complex pathologies by endoscopy, mainly where major bone decompression is required or in cases of reoperation, larger and more complex dural tears may occur that require repair [14].

In major dural tears, the historical recommendation has been to convert to open surgery and perform injury repair [13]. However, conversion is not without its own set of problems. Often, anesthesia must be converted into general anesthesia [15]. The patient may also not have been informed about the possibility of conversion to open surgery, which may lead to a disagreement between the patient and the surgeon [15].

In 2018, Shin et al. carried out the first report of a repair technique for dural injury using the full-endoscopic technique, the Youn technique [15]. In this technique, the surgeons aim for hermetic closure of the lesion using 6–0 Prolene sutures. To perform the procedure, no materials other than the usual set of endoscopic instruments are required. The authors used a double-needle thread passing through each side of the lesion, using special disk forceps during endoscopic surgery to manipulate the needle. The other end was outside the working cannula, and the suture knot was performed. To tighten the knot, a hollow curette for endoscopic surgery was used, passing one end of the wire through itself [15].

**Figure 3:**
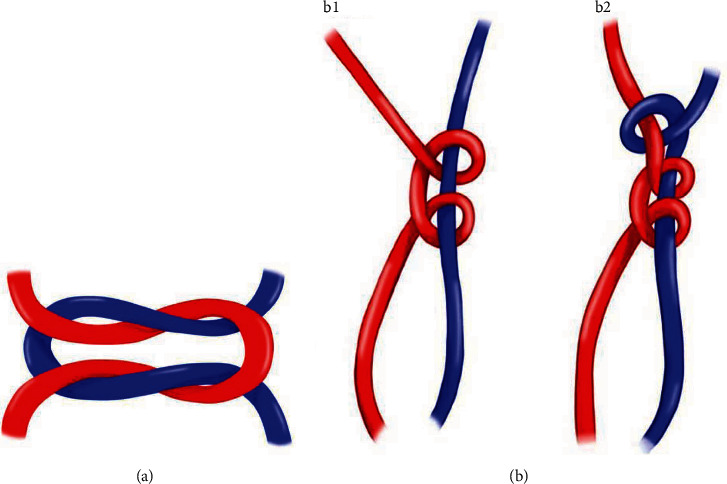
(a) Youn technique with 2-3 simple knots. (b) The Bergamaschi technique with the first sliding knot (b1) and the second locking knot (b2).

Our technique differs from Youn's technique by performing a reversing half-hitch alternating postsliding knot instead of a simple knot (Figure 3). A simple knot or square knot was used in freehand surgeries. The tension must be applied equally at both ends to tighten the knot. This can be a limitation or difficulty when there is limited space or in a deep cavity, as in endoscopic spine surgery, where greater tension applied to one of the wires can create a weak knot and lead to a greater chance of failure [23].

Unlike the traditional full-endoscopic technique, biportal endoscopic spine surgery involves the use of two spine approach portals, with the materials “floating” around the spine, using the concept of triangulation to navigate and address the pathology. Kim et al. [24] suggested a treatment protocol for dural sac injuries. Some occlusion and/or interposition techniques have been suggested for lesions smaller than 1 cm, using materials such as gelfoam and fibrin glue. In lesions larger than 1 cm, the suggested approach is to convert to open surgery and repair the lesion. Despite the benefit of having more than one approach portal, they did not describe any techniques for suturing the lesions.

In 2020, Park et al. [16] described a more detailed protocol for approaching dural injuries using the biportal technique. In lesions smaller than 4 mm, the protocol is to observe and keep the patient on bed rest for 24 h. In lesions between 4 mm and 12 mm in size, the recommended procedure is to apply a hard sealant and keep the patient in the hospital for 24 h of observation. In lesions larger than 12 mm, the protocol varies depending on the region of the lesion. For lesions in the dural sac (zone 2) or descending root (zone 3) with regular margins, the lesion is repaired with staples using special equipment, the patient remains hospitalized for a longer than 48 h, and an external lumbar shunt is considered. For lesions in the emerging root armpit (zone 1) or in zones 2 and 3 with irregular margins, the protocol is to convert to open surgery and perform primary repair of the lesion.

The failure of the primary suture of the dural lesion can vary from 5% to 9%, partly because of the holes created during the passage of the suture. In cases of very complex lesions, in which complete closure cannot be achieved or when there is suspicion of persistent CSF leakage through the holes in the passage of the threads, the suture should be complemented with some type of dural sealant [25–27].

Suturing a dural lesion using the full-endoscopic approach is difficult to perform and should be performed by experienced surgeons. Previous training on a cadaver or an anatomical model is encouraged to prepare specialists. With technical developments and new instruments that allow for the manipulation of the needle, full-endoscopic sutures can be used as the primary treatment in the future.

## 5. Conclusion

Endoscopic repair of dural lesions is challenging for spine surgeons. The Bergamaschi technique described here represents an advanced method of treatment capable of allowing more surgeons to treat dural injuries and for more complex pathologies to be treated with endoscopy without the need for conversion to open surgery.
